# Non-Mitogenic Fibroblast Growth Factor 1 Enhanced Angiogenesis Following Ischemic Stroke by Regulating the Sphingosine-1-Phosphate 1 Pathway

**DOI:** 10.3389/fphar.2020.00059

**Published:** 2020-03-03

**Authors:** Yuchi Zou, Jian Hu, Wenting Huang, Shasha Ye, Fanyi Han, Jingting Du, Mingjie Shao, Ruili Guo, Jingjing Lin, Yeli Zhao, Ye Xiong, Xue Wang

**Affiliations:** ^1^ The Frist Affiliated Hospital of Wenzhou Medical University, Wenzhou, China; ^2^ School of Pharmaceutical Sciences, Wenzhou Medical University, Wenzhou, China; ^3^ School of the First Clinical Medical Science, Wenzhou Medical University, Wenzhou, China; ^4^ Engineering Laboratory of Zhejiang Province for Pharmaceutical Development of Growth Factors, Biomedical Collaborative Innovation Center of Wenzhou, Wenzhou, China

**Keywords:** ischemic stroke, nmFGF1, angiogenesis, migration, S1P1 pathway

## Abstract

Ischemic strokes account for about 80% of all strokes and are associated with a high risk of mortality. Angiogenesis of brain microvascular endothelial cells may contribute to functional restoration following ischemia. Fibroblast growth factor 1 (FGF1), a member of FGF superfamily, involved in embryonic development, angiogenesis, wound healing, and neuron survival. However, the mitogenic activity of FGF1 is known to contribute to several human pathologies, thereby questioning the safety of its clinical applications. Here, we explored the effects and mechanism of action of non-mitogenic FGF1 (nmFGF1) on angiogenesis in mice after ischemia stroke and an oxygen-glucose deprivation (OGD)-induced human brain microvascular endothelial cells (HBMECs) injury model. We found that intranasal administration nmFGF1 significantly promoted angiogenesis in mice after stroke, and significantly increased the formation of matrigel tube and promoted scratch migration in a dose-dependent manner in OGD-induced HBMECs *in vitro*. However, the co-administration of an FGF receptor 1 (FGFR1)-specific inhibitor PD173074 significantly reversed the effects of nmFGF1 *in vitro*, suggesting that nmFGF1 functions *via* FGFR1 activation. Moreover, nmFGF1 activated sphingosine-1-phosphate receptor 1 (S1PR1, S1P1) in mice after stroke *in vivo*. S1P1 protein antagonist VPC23019 and agonist FTY720 were used to confirm that nmFGF1 promotes angiogenesis *in vitro* partially through the S1P1 pathway. OGD induced downregulation of S1P1 expression. The S1P1 antagonist VPC23019 blocked the stimulatory effects of nmFGF1, whereas the S1P1 agonist FTY720 exerted effects comparable with those of nmFGF1. Furthermore, PD173074 reversed the effect of nmFGF1 on upregulating S1P1 signaling. In conclusion, nmFGF1 enhanced angiogenesis in mice following stroke and OGD-induced HBMECs through S1P1 pathway regulation mediated *via* FGFR1 activation. This new discovery suggests the potential therapeutic role of nmFGF1 for the treatment of ischemic strokes.

## Introduction

Neurological disorders are the leading cause of disability-adjusted life-years (DALYs) worldwide and the second leading cause of deaths following cardiovascular diseases. Stroke, a global health problem, was the largest contributor to global neurological DALYs in 2016, and ischemic stroke is known to account for about 80% of all stroke cases (Markus, 2011; GBD 2016 Neurology Collaborators, 2019). However, current therapeutic approaches for ischemia stroke include intravenous thrombolysis, surgical thrombectomy, and neuroprotection (Wang et al., 2019). Recombinant plasminogen activator (rtPA), as the only approved thrombolytic agent for the acute ischemic stroke treatment, can only be administrated up to 6 hours after stroke onset, and may enhance the risk of hemorrhagic transformation. Therefore, development of an effective therapeutic strategy for stroke is urgent.

Neurorestorative progression in stroke is characterized with neurogenesis, angiogenesis, and synaptic plasticity, and is beneficial for functional recovery after stroke (Gopurappilly et al., 2011; Chen et al., 2019a). Angiogenesis, the formation of new capillaries from the pre-existing blood vessels, is observed in the penumbra of brain infarct region in animal model following stroke and in the brain of patients with stroke (Ruan et al., 2015). In the early stages of ischemia, capillaries proliferate, and damaged cells release a various factors to enhance angiogenesis, thus resulting in the formation of new blood vessels and increase of cerebral blood perfusion (Arenillas et al., 2007; Wang et al., 2019), which considered as a defense system that restores nutrient and oxygen supplies to the ischemic brain tissue (Tomanek and Schatteman, 2000; Chen et al., 2019a; Kanazawa et al., 2019). However, endogenous ischemic tissue stimulated angiogenesis may not adequately compensate for the acute impaired circulation (Nariai et al., 1994; Wang et al., 2019). Therefore, angiogenesis promotion exogenously is a promising treatment strategy for ischemic stroke, thereby, facilitating survival of brain tissue following ischemia stroke.

Sphingosine-1-phosphate (S1P), a bioactive lysophospholipid, and its receptors (S1PR1-5) highly expressed in various system including vascular, immune, nervous, and reproductive systems (Hla, 2004; Sartawi et al., 2017). S1P receptor 1 (S1PR1, S1P1) signaling is essential in endothelial cells (ECs) and regulates sprouting angiogenesis. S1PR1 is induced in angiogenic ECs and plays an important role in vascular development (Liu et al., 2000; Jung et al., 2012). On the contrary, blocking S1P signaling *via* S1PR1 inhibition may provide a new direction for antiangiogenic therapy to treat tumors (Liu et al., 2019; Rostami et al., 2019). It has been previously demonstrated that S1P1 activation could alleviate brain injuries in experimental ischemia stroke models such as transient middle cerebral artery occlusion (MCAO) (Hasegawa et al., 2010) and neonatal hypoxia-ischemia (Zhou et al., 2010). S1PR1 modulators involved in S1P1 signaling pathway improve microvascular circulation after thrombosis and exert beneficial roles in cerebral ischemia (Li et al., 2019). The above evidences indicated S1P1 activation could be considered as a good candidate target for ischemia stroke treatment.

Fibroblast growth factor 1 (FGF1) is a member of the FGF family and regulates various cellular processes such as angiogenesis, cell migration, cell differentiation, wound healing, and tube formation *via* binding to FGF receptors and heparin sulfate proteoglycans (Cheng et al., 2011; Wu et al., 2017; Kerr et al., 2019). However, the mitogenic activity of FGF1 is known to contribute to metastasis and tumorigenesis (Cronauer et al., 2003; Li et al., 2007). In the present study, the effects of non-mitogenic FGF1 (nmFGF1) derived from the deletion of the N terminal residues 1–27 of the full length wild-type FGF1 were investigated in an oxygen-glucose deprivation (OGD)-induced human brain microvascular endothelial cell (HBMECs) injury model and an ischemia and reperfusion-injured MCAO mouse model. Previous studies have demonstrated that FGF1 could induce neurogenesis and angiogenesis in rats after ischemic stroke (Cheng et al., 2011) and rescue hippocampal neurons from apoptotic death induced by ischemia (Cuevas et al., 1998). However, the mechanism underlying the action of FGF1 on angiogenesis is yet unclear. Here, we investigated whether exogenous nmFGF1 administration could promote angiogenesis through the S1P1 signaling pathway.

## Materials and Methods

### Reagents and Antibodies

NmFGF1 was produced in *Escherichia coli* and purified to be endotoxin free as previously described (Wu et al., 2005). The FGFR1-specific inhibitor PD173074 was purchased from Abcam (Cambridge, MA, USA). S1P1 antagonist VPC23019 was purchased from Cayman Chemical (Ann Arbor, MI). The primary antibodies applied in this study including anti-FGFR1 (No. ab824), anti-p-FGFR1 (No. ab59194), anti-S1P1 (No. ab11424), anti-CD31 (No. ab28364), and anti-β-Actin (No. ab8227) were purchased from Abcam (Cambridge, MA, USA), and anti-FGF1 (No. BM5544) was obtained from Bosterbio (Pleasanton, CA, USA). The secondary antibody used were goat anti-rabbit IgG H&L (HRP) (No. ab6721), and Alexa Fluor ^®^488-conjugated donkey anti-rabbit (No. ab150073) which were also purchased from Abcam (Cambridge, MA, USA). 3-(4,5-dimethylthiazol-2-yl)-2,5-diphenyl-tetrazolium bromide (MTT) and S1P1 agonist FTY720 were purchased from Sigma-Aldrich (St. Louis, MO, USA). EBM-2 medium was purchased from Lonza (Hopkinton, MA, USA). Matrigel matrix was obtained from Corning Inc (Tewksbury, MA, USA). All other chemicals were of analytical-reagent grade.

### Animals Grouping and Drug Administration

The experiments were conducted with male C57BL/6N mice (20–25 g), which were purchased from the Animal Center of the Chinese Academy of Sciences (Beijing, China). The animal use and care protocol conformed to the Guide for the Care and Use of Laboratory Animals from the National Institutes of Health and was approved by the Animal Care and Use Committee of Wenzhou Medical University.

**Figure 1:**
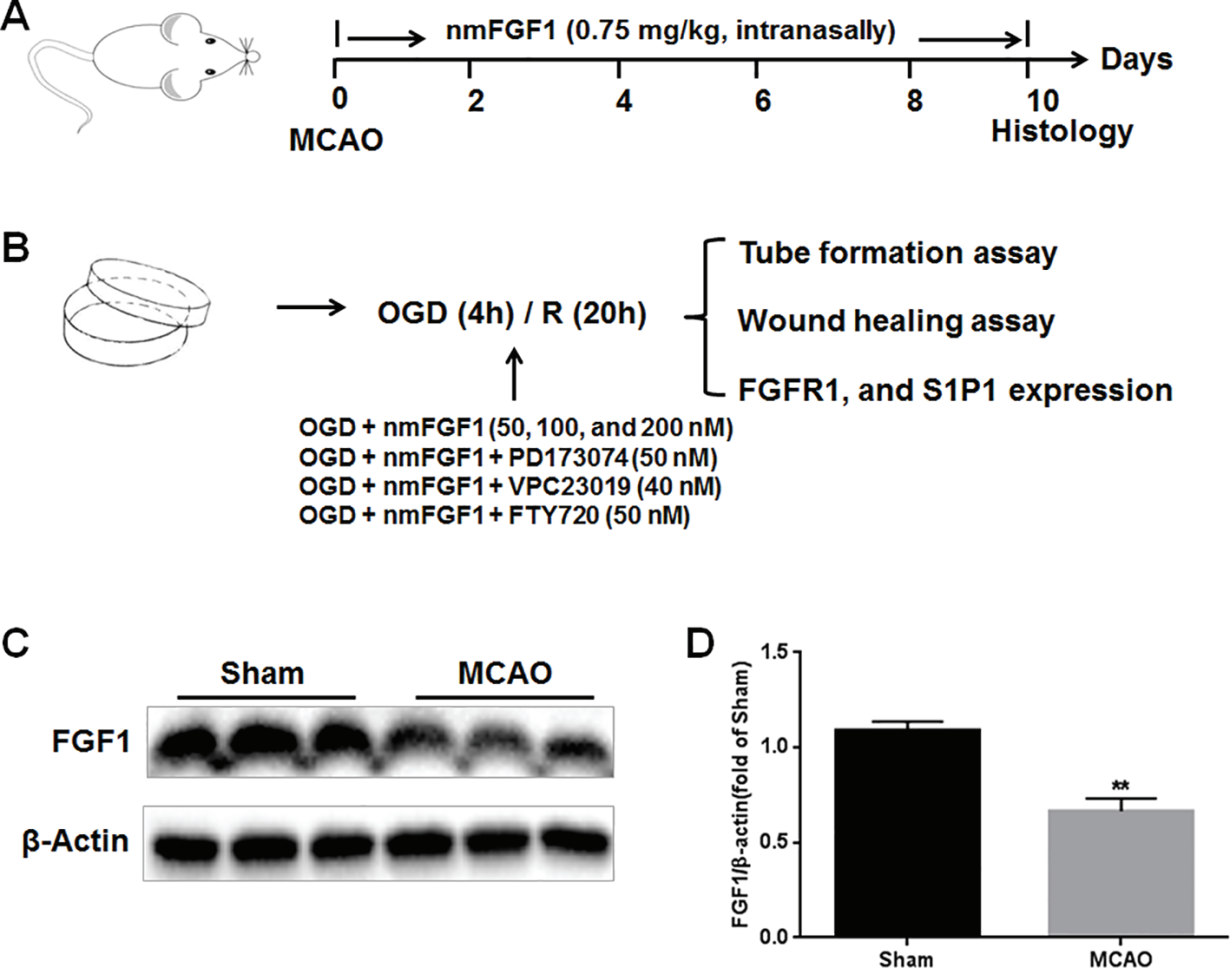
Illustration of experimental procedure. **(A)** Schematic illustration of animal experimental timeline showing the duration exogenous non-mitogenic fibroblast growth factor 1 (nmFGF1) administration after middle cerebral artery occlusion (MCAO) in adult mice and analysis. **(B)** Schematic illustration of cell experimental procedure after oxygen-glucose deprivation (OGD)/R and analysis. **(C)** Representative bands of endogenous FGF1 expression detected by western blot. **(D)** Densitometric analysis for the protein expression of FGF1. Data are expressed as means ± SEM (n = 5). **p < 0.01.

After MCAO, the mice were randomly divided into two groups, mice subjected to MCAO treated with saline (MCAO + vehicle group) and nmFGF1 treatment MCAO-induced mice (MCAO + nmFGF1 group). For MCAO + nmFGF1 group, nmFGF1 was intranasally administrated at a dose of 0.75 mg/kg, followed by MCAO surgery. NmFGF was intranasally administrated once daily for 10 consecutive days, then the brain of animals were prepared for further immunofluorescence analysis (**Figure 1A**).

### Transient Focal Cerebral Ischemia and Reperfusion Model Preparation

A transient focal cerebral ischemia mouse model was generated by MCAO followed by reperfusion, as previously reported (Zhang et al., 2017) with minor modifications. Briefly, the mice were anesthetized by isoflurane, and a surgical incision was made in the midline neck. The common carotid artery (CCA), external carotid artery (ECA), and internal carotid artery (ICA) were carefully separated and ligated. An appropriate nylon monofilament for MCAO (Guangzhou Jialin Co., Ltd, Guangzhou, China) coated with 1% poly-L-lysine was inserted from the right ECA to ICA and gently advanced to the origin of the middle cerebral artery (MCA) until some resistance occurred after the movement of the nylon monofilament. The nylon monofilament was gently removed after 1 hour of occlusion to allow reperfusion. During surgery, the regional cerebral blood flow (CBF) was measured by Laser-Doppler flowmetry (Perimed, Jarfalla, Sweden) based on the previous study (Jiang et al., 2016; Wang et al., 2018). And the mice that did not show a significant CBF reduction of at least 75% of baseline levels after MCA occlusion, or that died after ischemia stimulation or reperfusion excluded from further experimental study.

### Immunofluorescence Analysis

To evaluate cell proliferation, 5-ethynyl-2-deoxyuridine (EdU) labeling was performed using BeyoClickTM EdU-488 kit (C0071S, Beyotime, Shanghai, China) according to the manufacturer's protocol. From day 6 to day 10, all groups of mice were treated with EdU [50 mg/kg intraperitoneal (i.p.) injection in saline] once daily for 4 consecutive days. After 4 hours, the mice were transcardially perfused with ice-cold phosphate-buffered saline (PBS; 0.1 M), followed by 4% paraformaldehyde (dissolved in PBS). The brains were quickly excised for immunofluorescence staining. EdU staining was assessed with immunofluorescence.

Immunofluorescence staining was used to detect microvessel density. The whole brain tissue was post-fixed with 4% paraformaldehyde for 12 hours, embedded in paraffin, and cut into sections (5 µm thickness), followed by mounting on slides. The sections were treated with a primary antibody against CD31 (1:500 dilution) overnight at 4°C and then washed thrice with PBST at an interval of 5 minutes, followed by incubated with Alexa Fluor 488 or Alexa Fluor 647 donkey anti-rabbit/goat secondary antibodies (1:1000) for 1 hour at room temperature. Nuclear staining was performed with 4′,6-diamidino-2-phenylindole (DAPI) for 5 minutes. The stained sections were stored at 4°C. The immunostained sections were observed and imaged using a Nikon ECLPSE 80i fluorescence microscope (Nikon, Tokyo, Japan). Image-Pro plus 6.0 software (Bethesda MD, USA) was applied to analyze the images as previously described (Jiang et al., 2016). In brief, the density of the new blood vessel (EdU^+^ cells), blood vessel (CD31^+^ cells), or S1P1^+^ cells were automatically counted from three microscopic field randomly chosen from the peri-infarct regions in each three section from four animals of each group.

### HBMECs Culture and Treatment

HBMECs were purchased from Cell Systems Corporation (ACBRI376, Kirkland, WA, USA) and cultured in complete growth EBM-2 Medium with 10% FBS and penicillin/streptomycin (100 U/ml) in an atmosphere containing 5% CO_2_ at 37°C. To assess the effects of nmFGF1 on OGD-induced HBMECs and the possible underlying mechanisms, the cells were divided into the following groups: control group, OGD treatment group, OGD+nmFGF1-treated group, and OGD+nmFGF1+PD173074-treated group, OGD+nmFGF1+VPC23019-treated group, or OGD+nmFGF1+FTY720-treated group.

Control group is the HBMECs monolayers culture without OGD/R treatment. For the OGD/R treatment, the protocol was performed as described previously (Lin et al., 2018; Chen et al., 2019b). In brief, HBMECs cultured EBM-2 media were replaced with glucose free DMEM media, and then the cells cultured dish or plates were put in a specialized, humidified chamber (Heidolph, incubator 1000, Brinkmann Instruments, Westbury, NY) at 37°C, which contained an anaerobic gas mixture (90% N_2_, 5% H_2_, and 5% CO_2_), for 20 minutes to ensure anaerobic conditions, and for a further 4 hours incubation. Reperfusion was started by removing the culture dish or plates from the anaerobic chamber, and replacing the OGD medium with EBM-2 complete medium. Then the cells were allowed to reoxygenate in a regular incubator for 20 hours before further analysis. For nmFGF1-treated group, the cells were exposure to nmFGF1 containing medium with final concentration of 50, 100, and 200 nM during OGD/R. For OGD+ nmFGF1+PD173074-treated group, OGD+nmFGF1+VPC23019-treated group, or OGD+nmFGF1+FTY720-treated group, the cells were co-treated with nmFGF1 and PD173074 (50 nM), VPC23019 (40 nM), and FTY720 (50 nM) during OGD/R, respectively. Then these cells were applied for tube formation and wound healing assay. To explore the underlying mechanism of action, the FGFR1 and S1P1 expression level was assessed by western blot (**Figure 1B**).

### Cell Viability Assay

MTT assay was performed to determine cell viability as described previously (Huang et al., 2019). Briefly, HBMECs (1×10^4^ cells/well) were seeded in 96-well plates in complete growth EBM-2 medium and cultured overnight. After treatment, the medium was replaced with fresh medium containing 0.5 mg/ml MTT. After 3-hour incubation at 37°C, the medium was removed, followed by the formazan crystals dissolved in dimethyl sulfoxide. The absorbance was measured at 570 nm by a microplate reader with a reference setting of 630 nm.

### Tube Formation Assay

Tube formation is a classical *in vitro* model to assess the angiogenesis (Lu et al., 2019). To assess the effect of nmFGF1 on the angiogenic capacity of OGD-treated HBMECs, the tube formation assay was performed as described previously (Huang et al., 2019). In brief, Matrigel matrix was thawed at 4°C and added into each wells of a 48-well plate, and the plate was incubated 30 minutes to allow Matrigel solidification. The treated cells were seeded in the above Matrigel-coated plate (6×10^4^ per well) triplicate, and incubated in a 37°C incubator for 16 hours, after which the number of tubes in two random fields from each well was counted under a bright field microscope.

### Wound Healing Assay

Wound healing assay used to evaluate cell migration across a scratch gap *in vitro*, is also a classical *in vitro* model of angiogenesis (Lu et al., 2019). After OGD/R treatment, the smooth scratches was made perpendicular to the well plate by a 10 μl pipette tip, and the width of each scratch wound should be as close to identical as possible. Afterward, the treated cells were cultured in medium with 1% FBS for further 16 hour incubation. Bright field images of migration were taken and compared with images of the cells before scratching. The relative migration distance was quantified by ImageJ software. The value of gap closure was calculated by the following equation: % of gap closure = (W_0_−W_n_)/W_0_×%, where W_0_ is the gap width at 0 hour, and W_n_ is the gap width at 16 hours. Data were acquired from three independent experiments with each condition performed in triplicate.

### Western Blot Analysis

Ipsilateral hemisphere after MCAO with 1 hour reperfusion and HBMECs were lysed and proteins were extracted with RIPA lysis buffer (R0278, Sigma-Aldrich, St. Louis, MO, USA)) with 1% protease and phosphatase inhibitor cocktail (P1261, Solarbio, Beijing, China) at 4°C. The supernatant was collected after cell lysate centrifuge, and the protein concentration was measured by a Bradford Protein Assay Kit. An equivalent amount of protein (80 µg) was separated on 12% SDS-PAGE and transferred to PVDF membranes. The membranes were blocked with 5% nonfat milk at room temperature for 1 hour and were then blocked with primary antibodies, including those targeting FGFR1 (1:500), p-FGFR1 (1:500), S1P1 (1:1000), FGF1 (1:1000), and β-actin (1:1000), and then incubated with goat anti-rabbit IgG secondary antibody (1:10000). Finally, the immunolabeling was detected by an enhanced chemiluminescence (ECL; GE Healthcare) detection system according to the manufacturer's instruction. The intensity of the bands was quantified using Image Lab 3.0 software (Bio-Rad, Hercules, CA, USA).

### Statistical Analysis

The results are expressed as mean ± SEM. Three or more replicates were performed for each experiment. Statistical differences among data from multiple groups were analyzed by one-way ANOVA followed by Tukey's posttest using GraphPad Prism 7 (GraphPad Software, San Diego, CA, USA). P < 0.05 was considered statistically significant.

## Results

### nmFGF1 Promoted Angiogenesis in Ischemic Boundary Following Ischemia and Reperfusion

To evaluate the effects of exogenous FGF1 on angiogenesis after ischemia stroke, we firstly assessed the changes of endogenous FGF1 expression after MCAO by western blot analysis. As the result shown, the expression level of endogenous FGF1 was significantly reduced after MCAO with 1 hour reperfusion. (**Figures 1C, D**).

**Figure 2:**
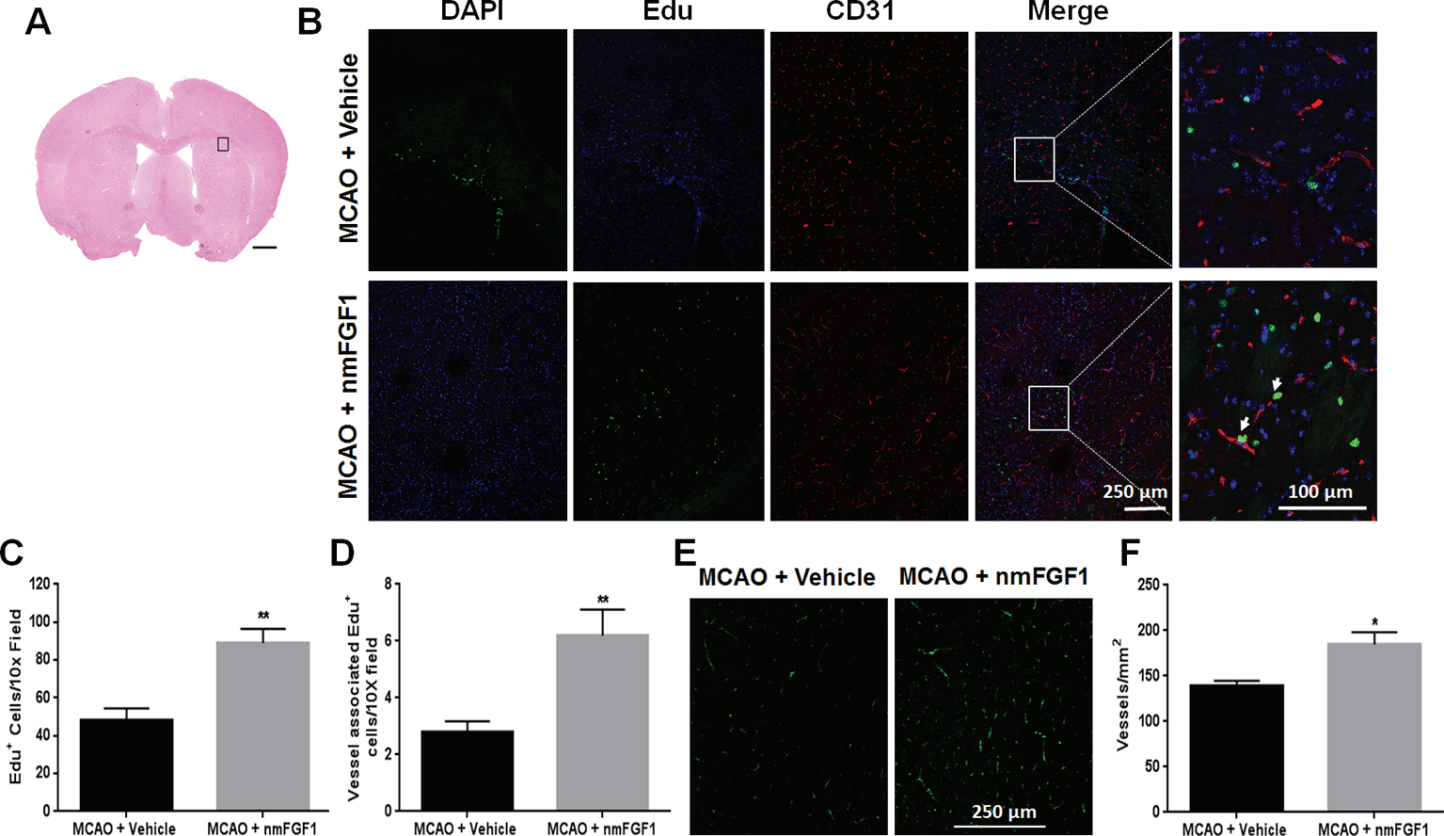
Non-mitogenic fibroblast growth factor 1 (nmFGF1) promoted angiogenesis in ischemic boundary following ischemia stroke. **(A)** A representative image of hematoxylin-eosin (HE) staining in a coronal brain section at 10 days after middle cerebral artery occlusion (MCAO). Box illustrates the peri-infarct areas from the corpus callosum where images in B were taken. **(B)** Representative immunofluorescence images of DAPI and EdU staining along with the staining for the vascular endothelium marker CD31 at 10 days after stroke. **(C)** Quantitative analysis of EdU- labeled cells in ischemic boundary following stroke. **(D)** Quantitative analysis of EdU^+^/CD31^+^ co-labeled cells in ischemic boundary following stroke. **(E)** Representative fluorescence image for vascular density analysis following staining with CD31 at 10 days after stroke. **(F)** Quantitative analysis of vascular density by CD31 labeling. Data are expressed as means ± SEM (n = 5). ^*^p < 0.05 and **p < 0.01 as compared with the MCAO + vehicle group.

EdU was intraperitoneally injected to mark proliferating cells, while CD31 was used as a marker of vessels. EdU- and CD31-immunoreactive cells are defined as markers of angiogenesis. To assess the effect of nmFGF1 on angiogenesis, the number of EdU-positive cells and vessel-associated EdU-positive cells was detected in peri-infarct corpus callosum at 10 days after reperfusion (**Figure 2A**). The number of EdU-positive cells (**Figures 2B, C**) and vessel-associated EdU-positive cells (**Figures 2B, D**) was significantly higher in nmFGF1 administration group than in MCAO + vehicle group. Vascular density was evaluated with CD31 immunofluorescence analysis. Quantitative analysis of CD31 labeling revealed that the number of vessels in the boundary region of ischemia at 10 days after reperfusion was significant higher in the mice treated with nmFGF1 than in those from MCAO + vehicle group (**Figures 2E, F**).

### nmFGF1 Promoted Angiogenesis *via* S1P1 Pathway Regulation

**Figure 3:**
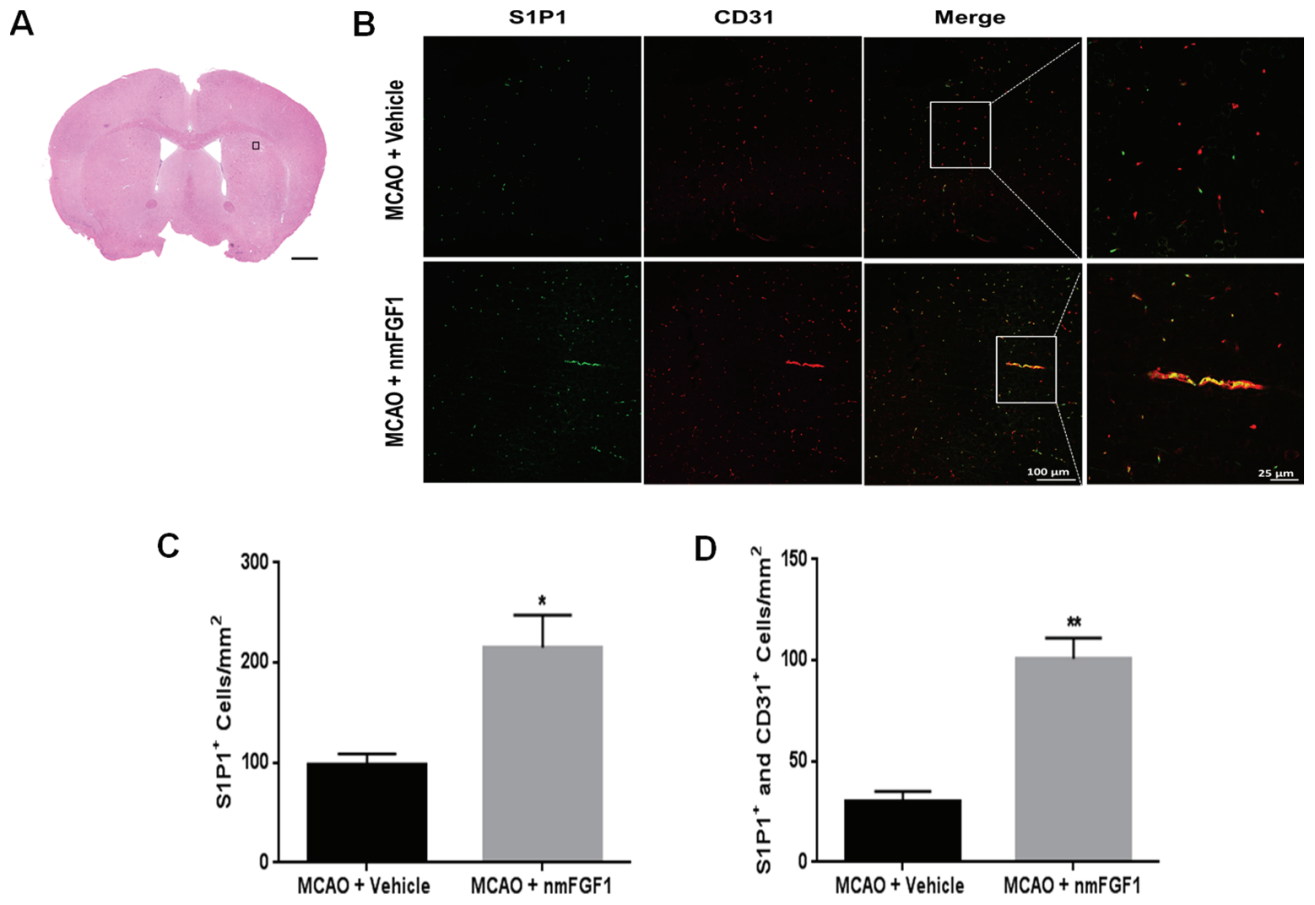
Non-mitogenic fibroblast growth factor 1 (nmFGF1) enhanced angiogenesis in ischemic boundary following ischemia stroke by S1P1 signaling pathway. **(A)** A representative image of HE staining in a coronal brain section at 10 days after middle cerebral artery occlusion (MCAO). Box illustrates the peri-infarct areas from the corpus callosum where images in B were taken. **(B)** Representative immunofluorescence images showing the co-localization of S1P1 and CD31 at 10 days after stroke. **(C)** Quantitative analysis of S1P1-positive cells in ischemic boundary following stroke. **(D)** Quantitative analysis of co-localization of S1P1 and CD31 at 10 days after stroke. Date are means ± SEM (n = 5). ^*^p < 0.05, and **p < 0.01 as compared to the middle cerebral artery occlusion (MCAO) + vehicle group.

S1P signaling *via* the cell surface receptor S1P1 is involved in several physiological processes, including angiogenesis, cell survival, and cell proliferation (Reinhard et al., 2017; Xie et al., 2017). nmFGF1 may enhance angiogenesis following stroke *via* S1P1 activation; hence, we evaluated the effect of nmFGF1 on S1P1 expression in peri-infarct corpus callosum at 10 days after reperfusion (**Figure 3A**). In comparison with the vehicle-treated MCAO-induced mice, the intranasal administration of nmFGF1-treated MCAO-induced mice showed a significant regulatory effect on the expression of S1P1 after 10 days (**Figures 3B, C**). The number of S1P1- and CD31-positive vascular ECs was markedly higher in these mice than in the vehicle-treated mice (**Figures 3B, D**). These results indicate nmFGF1 promoted angiogenesis through the activation of the S1P1 signaling pathway following stroke.

### nmFGF1 Enhanced Tube Formation and Wound Healing of HBMECs After OGD

**Figure 4:**
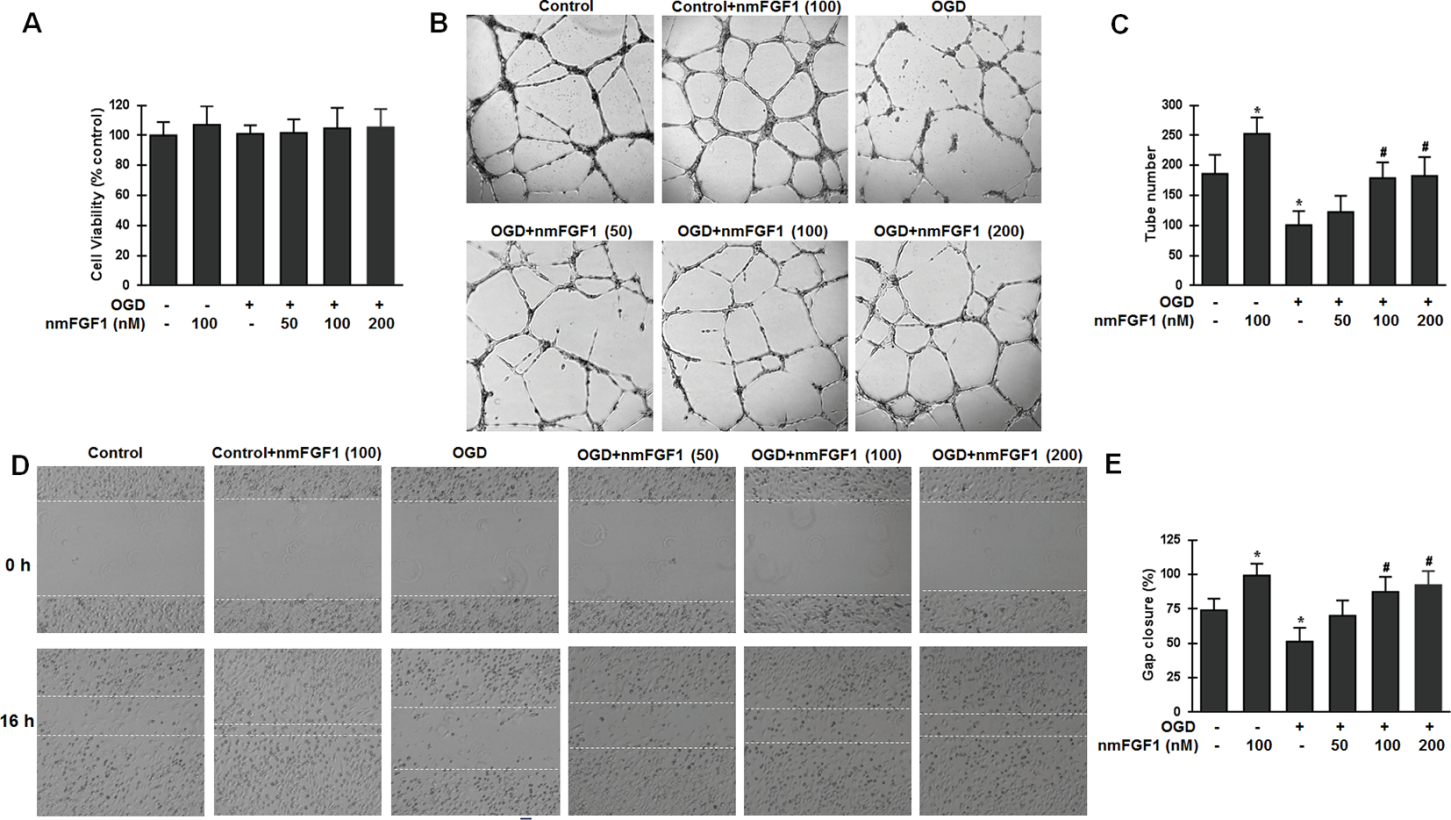
Non-mitogenic fibroblast growth factor 1 (nmFGF1) enhanced angiogenesis and wound healing in oxygen-glucose deprivation (OGD)-exposed HBMECs without affecting viability. **(A)** OGD and three concentrations of nmFGF1 (50, 100, and 200 nm) had no effect on cell viability. **(B)** Representative images to study the effect of OGD exposure and nmFGF1 treatment on tube formation ability of cells. **(C)** Quantification of the effects of nmFGF1 on the number of capillary-like tubes in OGD-treated cells. **(D)** Representative images to study the effect of nmFGF1 on wound healing in OGD-stimulated cells. **(E)** Quantification of the effects of nmFGF1 on wound closure in OGD-treated cells. Date are expressed as means ± SEM (n = 6). ^*^p < 0.05 as compared to the control group, ^#^p < 0.05 as compared to the OGD-treated group.

To confirm our hypothesis that nmFGF1 promotes angiogenesis after stoke, OGD/R-induced HBMECs were used. We tested whether nmFGF1 imparts protection against OGD/R-induced cytotoxicity by assessing the viability of HBMECs with the MTT assay. To induce OGD/R, HBMECs were subjected to OGD for 4 hours, followed by 20 hours of reoxygenation treatment. In nmFGF1 treatment group, HBMECs were exposed to nmFGF1 at 50, 100, and 200 nM concentrations during OGD/R. The effect of nmFGF1 (100 nm) on cell proliferation and tube formation under normal oxygen conditions was also assessed. As a result, we found that 100 nM nmFGF1 had no effect on cell proliferation under normal oxygen conditions, and OGD/R exhibited no significant effects on cell viability. nmFGF1 at 50, 100, and 200 nM concentrations did not markedly change the viability of cells under OGD conditions (**Figure 4A**), indicating that the protective effect of nmFGF1 on cell angiogenesis after OGD/R was not mediated through an increase in cell viability. To elucidate the effect of nmFGF1 on angiogenesis after OGD, tube formation assay was performed. The results demonstrate that nmFGF1 at a dose of 100 nm significantly increased the number of tubes and tubular structures as compared with the control group comprising cells cultured under normal conditions (complete medium). However, OGD-induced HBMECs showed lower angiogenic capacity than the control cells. Interestingly, nmFGF1 at 100, and 200 nM could markedly rescue the angiogenesis of HBMECs after OGD, while low dose (50 nM) nmFGF1 treatment failed to impart obvious protection (**Figure 4B**). Quantitative analysis revealed that nmFGF1 (100 and 200 nM) significantly increased the tube formation ability of HBMECs with similar angiogenesis efficacy (**Figure 4C**).

EC migration is another classical method for vascular angiogenesis assessment. The effects of nmFGF1 at different concentrations (50, 100, and 200 nM) on cell migration were analyzed by the scratch wound healing assay. As a result, nmFGF1 at a dose of 100 nm could significantly increase EC migration as compared with the control treatment. While OGD condition induced migration defects, nmFGF1 (100 and 200 nM) treatment could obviously facilitate wound healing. Quantitative analysis revealed that 100 and 200 nM nmFGF1 exerted similar effects; however, 50 nM nmFGF1 had no significant effects on cell migration (**Figures 4D, E**). Based on the above results, 100 nM nmFGF1 was used in the subsequent experiment to study the underlying mechanism of action.

### nmFGF1 Induced Angiogenesis in HBMECs After OGD *via* Activation of FGFR1 Signaling

**Figure 5:**
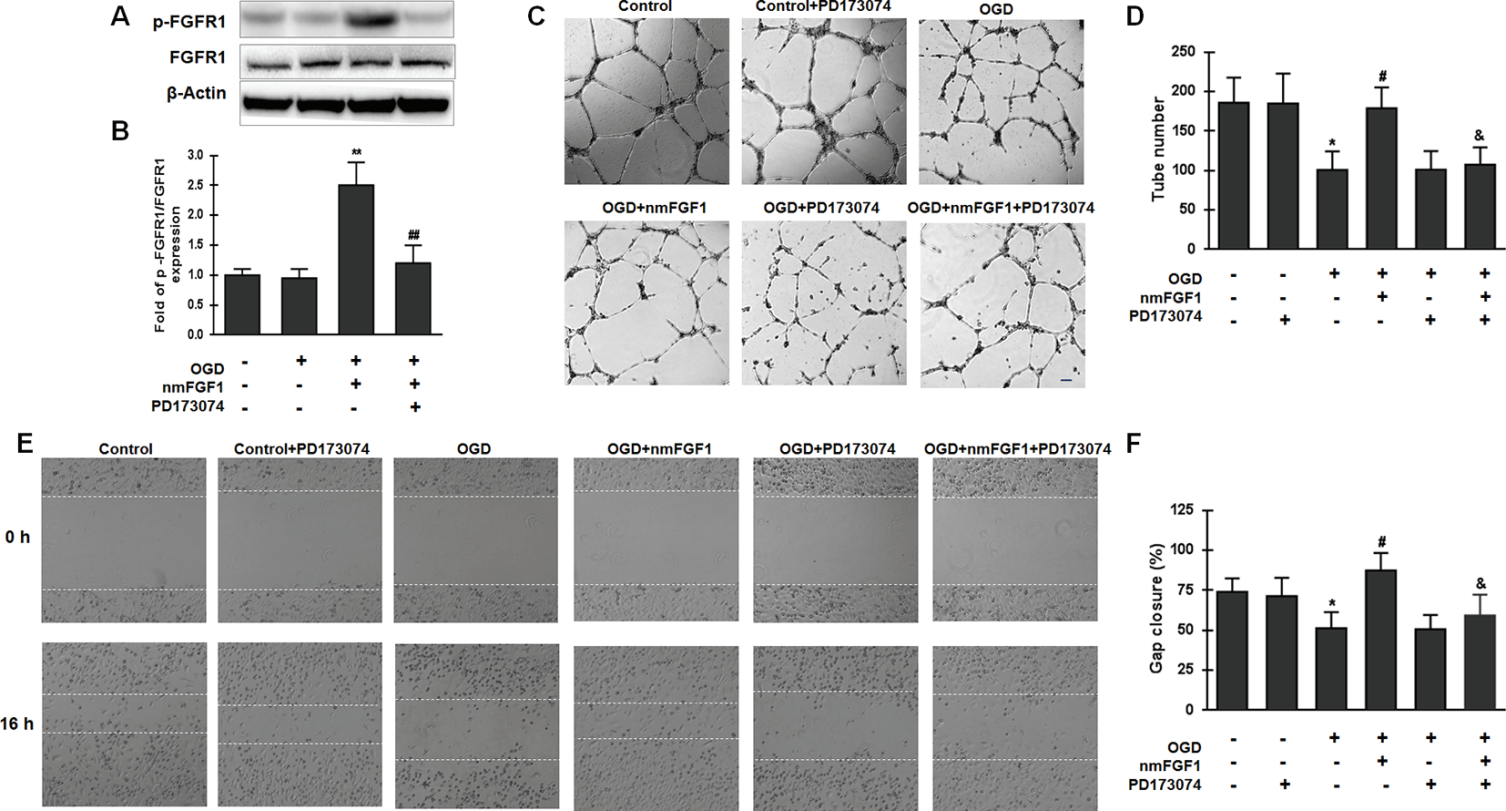
Non-mitogenic fibroblast growth factor 1 (nmFGF1) promoted angiogenesis through FGFR1 activation. **(A)** Representative image of western blot analysis for FGFR1 and p-FGFR1 expression. **(B)** Quantification of relative protein levels of p-FGFR1 and FGFR1. **(C)** Representative images for the analysis of the effects of cotreatment with the FGFR1 inhibitor PD173074 and nmFGF1 (100 nM) on tube formation ability of oxygen-glucose deprivation (OGD)-treated HBMECs. **(D)** Quantification of the effects of nmFGF1 and PD173074 cotreatment on the number of capillary-like tubes in OGD-treated cells. **(E)** Representative images showing the effects of PD173074 and nmFGF1 (100 nM) co-administration on wounding healing in OGD-stimulated cells. **(F)** Quantification of the effects of PD173074 and nmFGF1 (100 nM) co-administration on gap closure in OGD-treated cells. Date are means ± SEM (n = 6). ^*^p < 0.05 compared to control group, ^#^p < 0.05 as compared to the OGD-treated group, ^&^p < 0.05 as compared to the nmFGF1 treated group.

FGFs and their receptors (FGFRs) are critical in many biological processes, including angiogenesis, wound healing, and tissue regeneration (Sun et al., 2017; Xue et al., 2018). To investigate whether FGFR1 signaling is involved in angiogenesis mediated by nmFGF1 in HBMECs after OGD, cells were treated with the combination of FGFR1 inhibitor PD173074 (10 nM) and nmFGF1, and the activation of FGFR1 was detected by measuring the phosphorylation of FGFR1 (p-FGFR1) using western blotting. As a result, we found that the ratio of p-FGFR1/FGFR1 was not changed after OGD treatment; however, nmFGF1 significantly upregulated the ratio of p-FGFR1/FGFR1. As expected, PD173074 prevented the increase in the ratio of p-FGFR1/FGFR1 (**Figures 5A, B**).

We also assessed angiogenesis and migration ability and found that PD173070 administration alone had no effects on the tube formation ability of HBMECs under normal culture conditions. On the other hand, 100 nM nmFGF1 treatment significantly improved the tube formation ability of OGD-induced cells, while cotreatment with nmFGF1 and PD173074 significantly suppressed this proangiogenic effect. PD173074 alone had no effect on angiogenesis of OGD-induced cells (**Figures 5C, D**). Consistent with the effect of nmFGF1 on HBMECs tube formation ability, treatment with 100 nM nmFGF1 significantly promoted HBMECs migration, which was significantly suppressed in the presence of PD173074; however, PD173074 alone had no effect on cell migration (**Figures 5E, F**). These results suggest that the protective effects of nmFGF1 on tube formation and wound healing of OCD-induced HBMECs could be dependent on FGFR1 signaling activation.

### nmFGF1 Induced Angiogenesis in HBMECs After OGD/R by Upregulating S1P1 Expression

**Figure 6:**
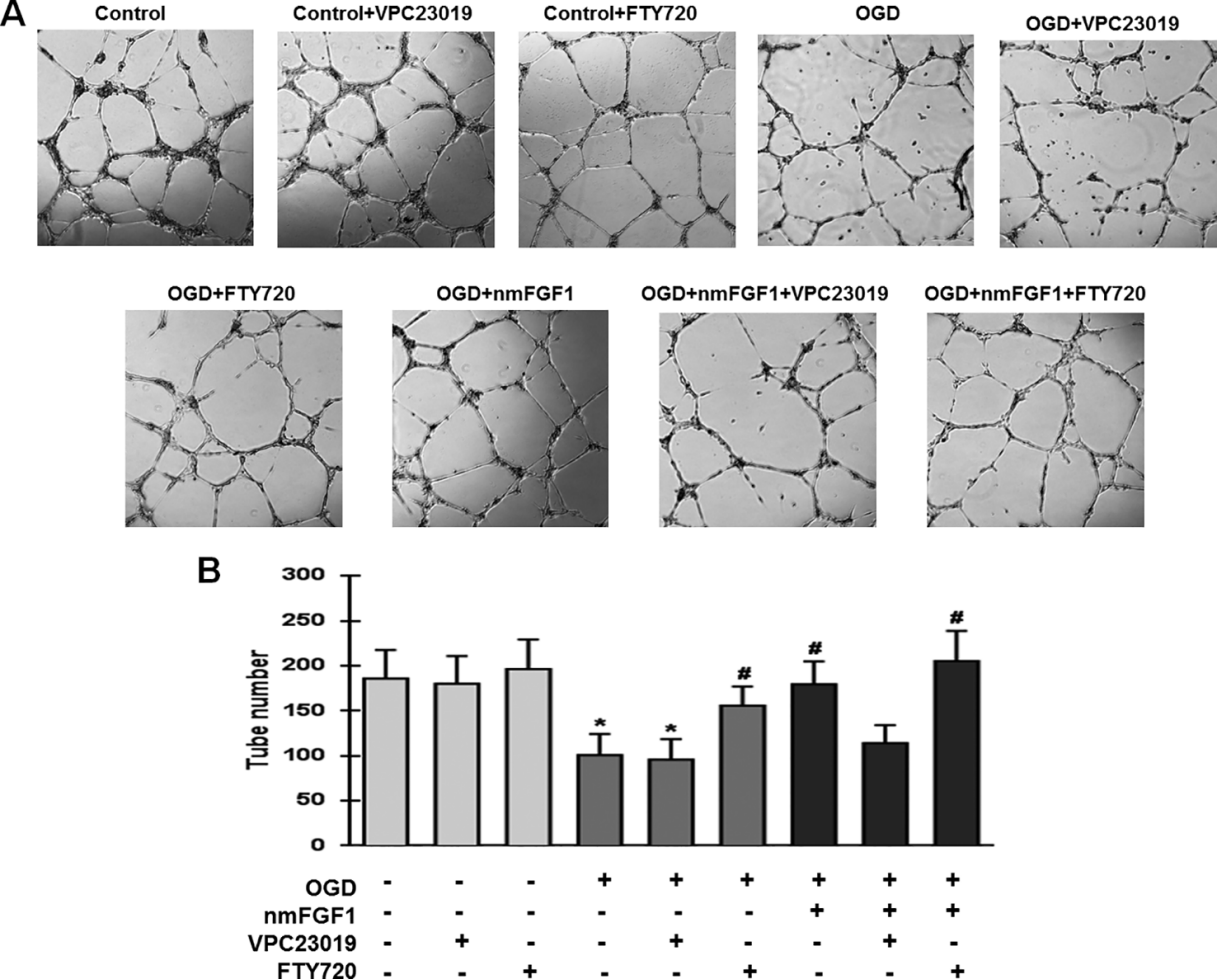
Non-mitogenic fibroblast growth factor 1 (nmFGF1) promoted angiogenesis of oxygen-glucose deprivation (OGD)-exposed HBMECs *via* S1P signaling pathway. **(A)** Representative images showing the tube formation ability of OGD-exposed cells treated with the S1PR1 inhibitor VPC23019 (40 nM) and agonist FTY720 (50 nM) in the presence or absence of nmFGF1. **(B)** Quantification of the effects of the S1P1 inhibitor VPC23019 (40 nM) and agonist FTY720 (50 nM) in the presence or absence of nmFGF1 on tube formation ability. Date are means ± SEM (n = 6). ^*^p < 0.05 as compared to the control group, ^#^p < 0.05 as compared to the OGD-treated group.

To confirm whether S1P1 is necessary to mediate the angiogenesis effects of nmFGF1 on HBMECs, the cells were treated with the S1P1-specific inhibitor VPC23019 (40 nM) and agonist FTY720 (50 nM) in the presence of nmFGF1 during OGD/R (Lin et al., 2018), and tube formation and scratch wound healing assays were performed. The results show that VPC23019 and FTY720 administration alone had no effect on the tube number in control cells. While VPC23019 treatment alone failed to affect the tube formation ability of HBMECs subjected to OGD/R, the treatment with FTY720 markedly enhanced the number of tubes. VPC23019 significantly inhibited the tube formation ability of nmFGF1 in HBMECs during OGD/R, and FTY720 in combination with nmFGF1 increased the number of tubes (**Figures 6A, B**).

**Figure 7:**
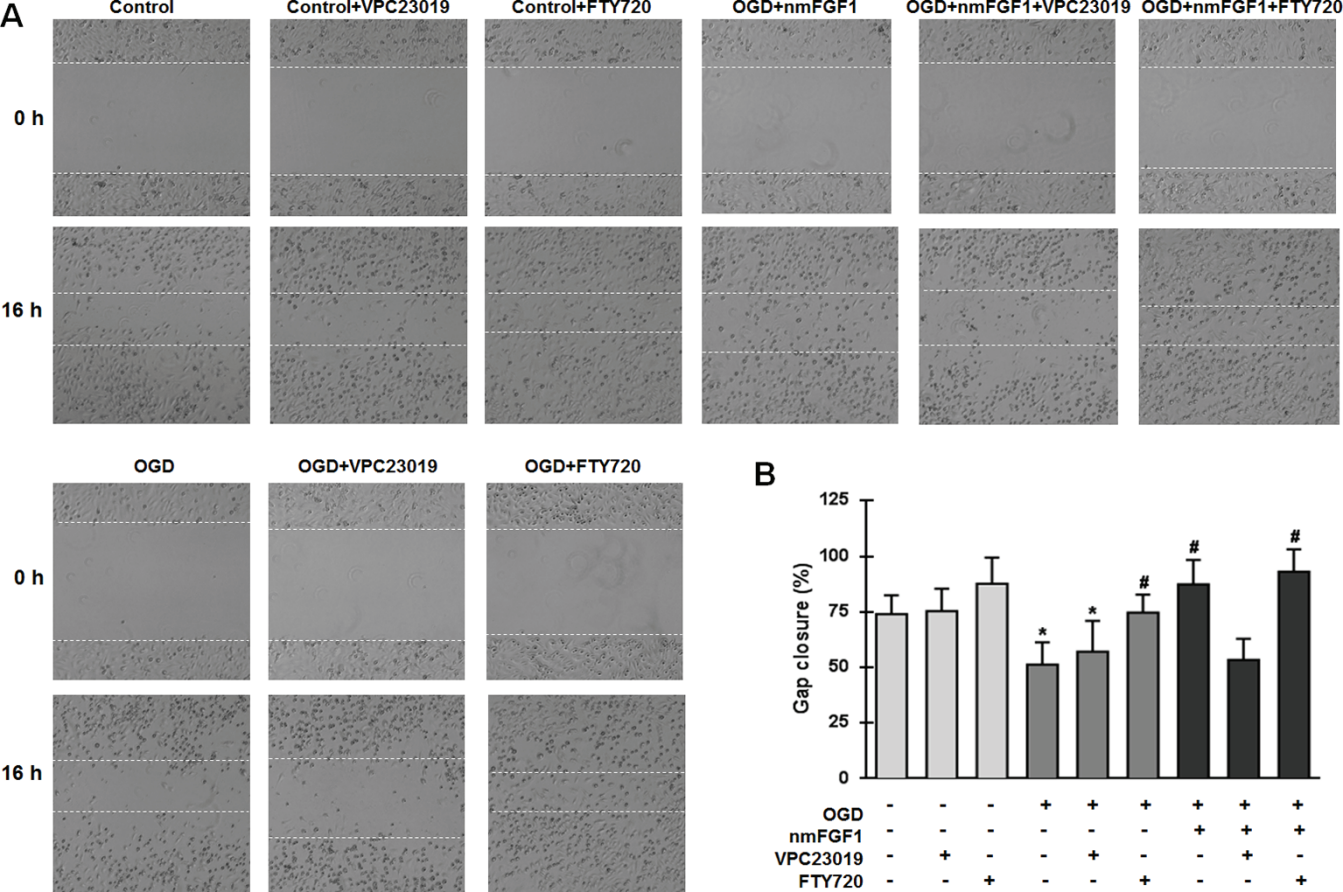
Non-mitogenic fibroblast growth factor 1 (nmFGF1) promoted wound repair in oxygen-glucose deprivation (OGD)-exposed HBMECs *via* S1P signaling pathway. **(A)** Representative images showing the migration of OGD-exposed cells treated with the S1P1 inhibitor VPC23019 (40 nM) and agonist FTY720 (50 nM) in the presence or absence of nmFGF1. **(B)** Quantification of the effects of the S1P1 inhibitor VPC23019 (40 nM) and agonist FTY720 (50 nM) in the presence or absence of nmFGF1 on wound healing. Date are means ± SEM (n = 6). ^*^p < 0.05 compared to control group, ^#^p < 0.05 as compared to the OGD-treated group.

We examined the wound healing ability of cells after treatment with VPC23019 and FTY720. While nmFGF1 significantly promoted the migration of OGD/R-induced cells in the scratch wound healing assay, VPC23019 obviously reversed this effect. S1P1 agonist FTY720 alone or in combination with nmFGF1 significantly increased the migration ability of cells. VPC23019 alone had no significant effects on the migration of HBMECs in both normal conditions and OGD/R environment (**Figures 7A, B**). These results suggest that nmFGF1 could rescue the OGD/R-induced S1P1 downregulation to enhance the tube formation and migration abilities of HBMECs under OGD/R conditions.

### nmFGF1 Rescued the OGD/R-Induced Downregulation of S1P1 Mediated by FGFR1 Activation

**Figure 8:**
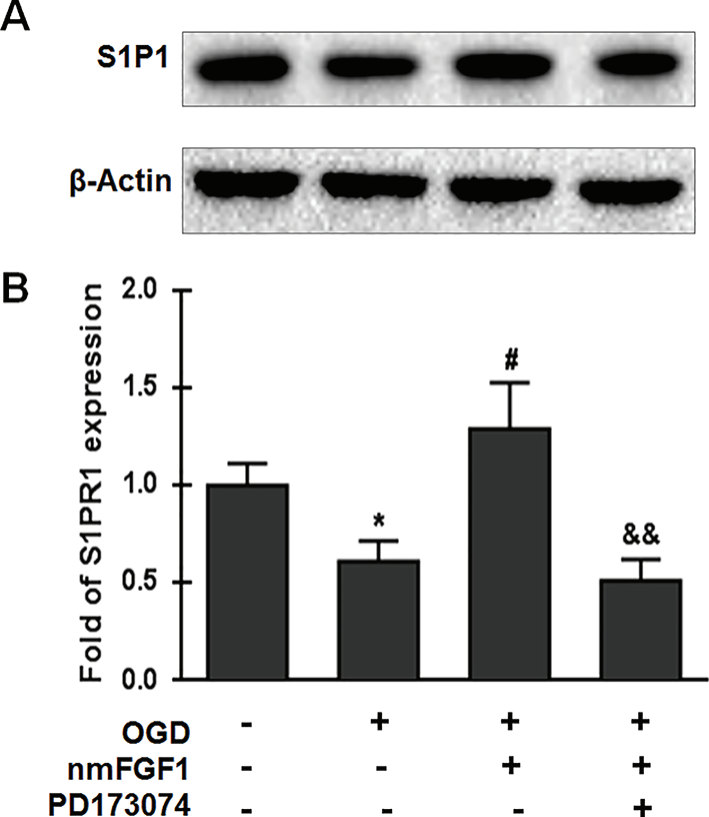
Non-mitogenic fibroblast growth factor 1 (nmFGF1) rescued oxygen-glucose deprivation (OGD)/R-induced downregulation of S1P1 expression by FGFR1 activation. **(A)** Representative western blots for S1P1 expression. **(B)** Quantification of the relative expression level of S1P1. Date are expressed as the means ± SEM (n = 4). ^*^p < 0.05 as compared to the control group, ^#^p < 0.05 as compared to the OGD-treated group, ^&&^p < 0.01 as compared to the nmFGF1-treated group.

S1P signaling through its cell surface receptor S1P1 is involved in several physiological processes, including angiogenesis, cell survival, and cell proliferation (Reinhard et al., 2017; Xie et al., 2017). We investigated whether nmFGF1 affects S1P1 expression. HBMECs were subjected to OGD for 4 hours and reoxygenation for 4 hours, and were simultaneously treated with nmFGF1. The protein expression of S1P1 was measured with western blotting. As a result, we found that S1P1 protein level was significantly suppressed by OGD/R. However, nmFGF1 administration obviously rescued S1P1 expression (**Figures 8A, B**). Moreover, FGFR1-specific inhibitor PD173074 markedly reversed the nmFGF1-mediated increase in S1P1 expression level (**Figures 8A, B**). This result suggests that nmFGF1 upregulates S1P1 expression through FGFR1 activation.

## Discussion

Angiogenesis is the formation of new microvessels from the pre-existing vessels (Ruan et al., 2015; Hatakeyama et al., 2020). Stroke is the primary cause of disability, owing to the limited ability to restore the damaged brain tissue (Nih et al., 2018; Rust et al., 2019). After ischemia stroke injury, inflammatory response coupled with limited angiogenesis and neuronal growth leads to functional deficits. A model of cerebral ischemia-reperfusion was established to investigate the pathophysiology of stroke. MCAO model is widely used for the induction of focal cerebral ischemia (Cotrina et al., 2017). OGD serves as an experimental approach to replicate ischemic stroke (Chen et al., 2019a). Angiogenesis is essential for brain tissue repair following ischemia stroke, as it may increase blood flow containing oxygen and metabolic nutrients to reach the affected brain regions (Hatakeyama et al., 2020). Several studies have focused on angiogenic therapy development for the recovery of injured brain following ischemia. In the current study, we explored the effects of nmFGF1 on angiogenesis in an MCAO ischemia mouse model and OGD-induced HBMECs *in vitro*. We found that nmFGF1 improved the angiogenesis in cerebral ischemia mice at day 10 after MCAO. We also demonstrate that nmFGF1 promoted angiogenesis and wound healing in OGD-induced HBMECs. In addition, we show for the first time that the angiogenesis effects of nmFGF1 were abolished by FGFR1- and S1P1-specific inhibitor, indicating that these effects were mediated through the activation of S1P1 signaling pathway and FGFR1.

FGF1 is unable to freely cross the brain upon systemic administration because of its filtration by the blood-brain barrier (BBB). However, intranasal administration may facilitate its direct entry into the central nervous system without exerting any adverse effects (Cheng et al., 2011). Previous studies have shown that the intranasal administration of FGF1 could enhance angiogenesis after stroke (Cheng et al., 2011). However, the mitogenic activity of FGF1 may contribute to several pathologies or metastasis and tumorigenesis (Cronauer et al., 2003; Li et al., 2007). For the safe application of FGF1, we applied nmFGF1 to mice after stroke and HBMECs after OGD/R. As expected, nmFGF1 treatment could enhance angiogenesis after ischemia injury in mice and promote angiogenesis and wound healing in OGD/R-induced HBMECs.

Although several studies have reported the protective effects of FGF1 on angiogenesis, the underlying mechanism of action on angiogenesis is unclear. FGFR1 is expressed in the brain (Wang et al., 2017; Huang et al., 2019), and its activation may protect the BBB in OGD/R-induced HBMECs (Lin et al., 2018). Cotreatment with the FGFR1 inhibitor PD173074 reversed the positive effects of nmFGF1 on migration and angiogenesis, indicating that FGFR1 activation induced migration and angiogenesis of HBMECs stimulated with OGD/R. In addition, S1P and its five receptors (S1PR1-5) are widely expressed in several systems, including the vascular, nervous, and reproductive system (Sartawi et al., 2017). S1P signaling pathway has been implicated in angiogenesis and is required for vascular stabilization. Dysfunction in the S1P-S1PR signaling pathway induces various vascular defects (Fischl et al., 2019; Obinata and Hla, 2019). The temporal profile of changes of the S1P1 expression in the early phase after transient MCAO was documented by Hasegawa et al. (2013), that the expression of S1P1 was significantly decreased in the infarct cortex but preserved in the peri-infarct cortex at 24 hours after MCAO. It has been reported that S1P1 expression on ECs of leptomeningeal arteries and capillaries upregulated early after permanent MCAO, peaking at 6 hours, whereas a significant increase in the expression of S1P1 in neurons was observed from 24 hours later (Iwasawa et al., 2018). S1PR modulators exerted protective effects in preclinical and clinical studies following ischemic stroke. In addition, these agents may improve microvascular circulation in cerebral ischemia (Lin et al., 2018). It has been reported that S1P1 agonist LAS1238 significantly reduced infarct volume of mice model after ischemia/reperfusion, indicating S1P1 is potential target for ischemic stroke treatment (Brait et al., 2016). Besides, a previous study reported that the activation of S1P signaling could protect the BBB after exposure of HBMECs to OGD/R (Lin et al., 2018). However, the effects of nmFGF1 on S1P signaling pathway are unknown. In our current study, we confirmed nmFGF1 significantly increased S1P1 expression in the ECs indicated by colocation of CD31 and S1P1 by immunofluorescence assay after 10 days following MCAO, that was consisted with previous study (Iwasawa et al., 2018). We treated OGD/R-induced HBMECs with S1P1 antagonist VPC23019 and S1PR1 agonist FTY720 in combination with nmFGF1. VPC23019 treatment alone had no effect on tube formation, while FTY720 markedly enhanced the number of tubes. In the presence of VPC23019, the tube formation ability of HBMECs induced by nmFGF1 was significantly inhibited during OGD/R, and FTY720 in combination with nmFGF1 increased the tube number. nmFGF1 exhibited similar angiogenesis ability with S1PR1 agonist FTY720, that was consisted with previous study (Shang et al., 2020). Although we did not perform the experiment to observe the effects of nmFGF1 on neuroprotection after stroke, combined with the previous study that FGF1 enhanced neurogenesis after stroke (Cheng et al., 2011), we can speculate one potential mechanism of nmFGF1 neuroprotection was that enhanced angiogenesis might be partially due to improvements in the microenvironment of vessel growth. As the data shown, PD173074 also inhibited the positive effects of nmFGF1 on S1P1 signaling. Taken together, nmFGF1 can promote angiogenesis through S1P signaling pathway mediated by FGFR1 activation.

## Conclusion

Our study provides the first evidence that intranasal nmFGF1 administration enhanced angiogenesis *via* S1P1 signaling pathway following stroke and highlights the need to better understand the mechanism underlying nmFGF1 function in ischemia protection. Taken together, these results suggest that nmFGF1 could be considered as potential angiogenic agent for ischemic stroke treatment.

## Data Availability Statement

The datasets generated for this study are available on request to the corresponding authors.

## Ethics Statement

All animal use and care protocols conformed to the Guide for the Care and Use of Laboratory Animals from the National Institutes of Health and were approved by the Animal Care and Use Committee of Wenzhou Medical University.

## Author Contributions

XW and YX designed the experiment and wrote the manuscript. YuZ, JH, WH, and SY performed the animal experiments. FH, JD, RG, and MS performed the cells experiments. JL and YeZ analyzed the data. All the authors read and approved the final version of the manuscript.

## Funding

This work was supported by Wenzhou Municipal Science and Technology Bureau Project (Y20170224 and Y20170686), Natural Science Foundation of Zhejiang Province (LQ19H090012), and Medical and Health Science and Technology Program of Zhejiang Province (2018KY505).

## Conflict of Interest

The authors declare that the research was conducted in the absence of any commercial or financial relationships that could be construed as a potential conflict of interest.

The handling editor declared a shared affiliation, though no other collaboration, with the authors at time of review.
